# Displacement- versus velocity-encoding: tracking regional myocardial motion using MR imaging

**DOI:** 10.1186/1532-429X-18-S1-P5

**Published:** 2016-01-27

**Authors:** Kai Lin, Jeremy Collins, Varun Chowdhary, Michael Markl, James C Carr

**Affiliations:** grid.465264.7Radiology, Northwestern University, Chicago, IL USA,

## Background

Magnetic resonance (MR) imaging has become an important diagnostic tool for the detection of impaired myocardial motion, which has been considered as a common consequence of most ischemic or non-ischemic heart diseases. Both cine DENSE and TPM have been independently adopted to delineate abnormal regional myocardial motion patterns for the estimation of the progression of cardiovascular diseases or for the evaluation of individual cardiovascular responses to treatments. However, although the similarities and differences between cine DENSE and TPM are well understood from a technical point of view, the interchangeability of those two imaging techniques in cardiovascular risk stratification is still not fully understood. The aim of the present study was to test the hypothesis that clinical metrics derived from cine DENSE and TPM are correlated for characterizing regional myocardial motion.

## Methods

This study complied with HIPAA regulations. Thirteen healthy volunteers and 4 asymptomatic recipients of heart transplant (HTx) were recruited following the approval of the institutional review board (IRB). For each participant, two-dimensional (2D) cine DENSE and TPM were performed at basal, midventricular and apical locations at the left ventricle (LV) with the short-axis view. DENSE-derived myocardial metrics, including peak radial strain (Err), circumferential strain (Ecc), first principal strain (E1), second principal strain (E2) and twist were measured and correlated with TPM-derived indices, including peak radial velocity (Vr), circumferential velocity (VΦ) and time to peak (TTP) in systole and diastole using Pearson correlation coefficient (r).

## Results

**Figure 1:**
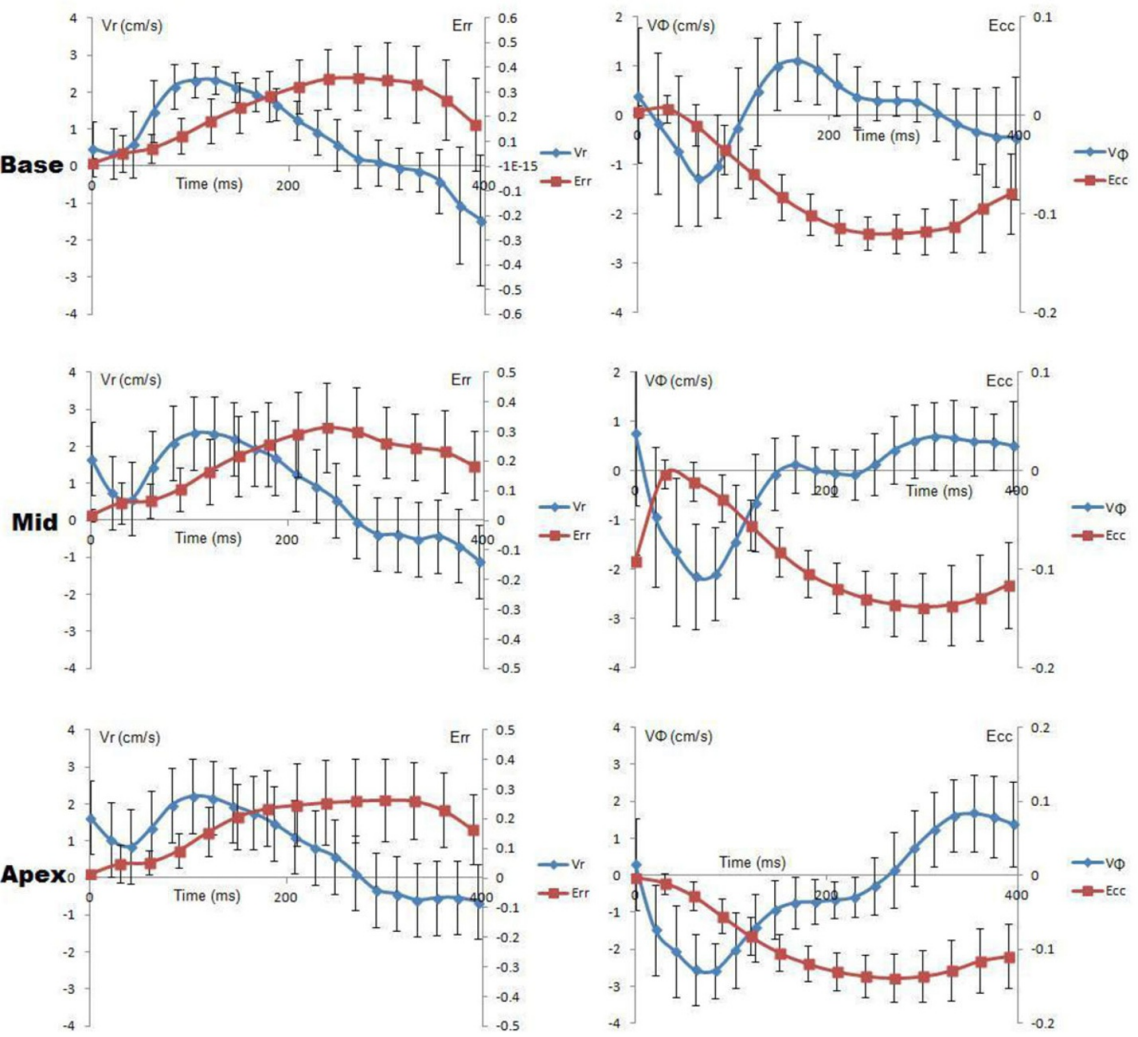
**Time curves for variations of Vr, Vφ, Err and Ecc during a cardiac cycle**. The data were averaged over 17 participants. The strains vary differently from the velocities. A certain velocity may correspond to different strains in various time points. However, peak velocities seem to appear prior to peak strains. *Only data in systole are included.

**Figure 2:**
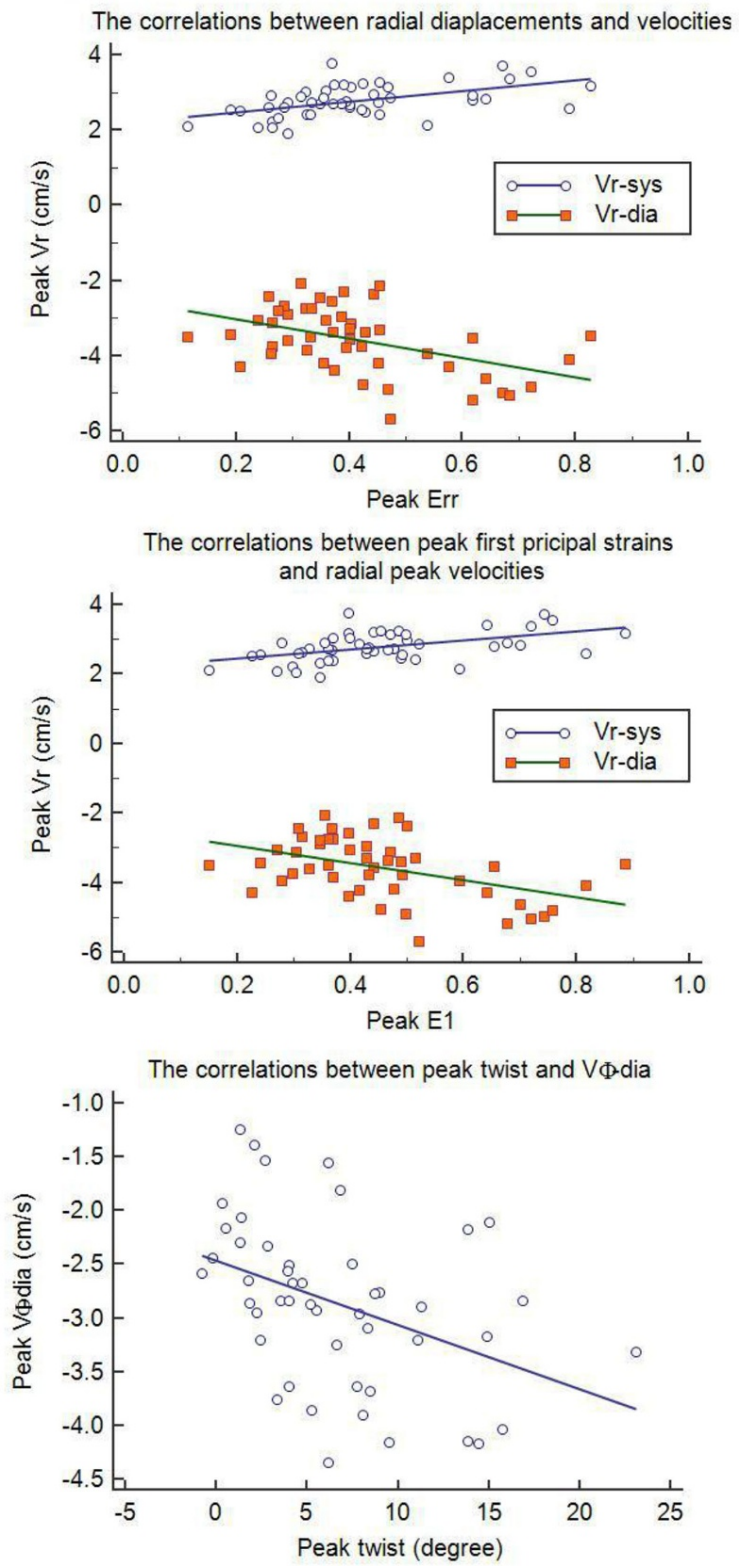
**Correlations between peak strains and velocities**.

DENSE and TPM data were successfully acquired in 17 participants and resulted in 51 data points (cross-sectional LV slices) for comparisons. On a per-slice basis, peak Err had moderate correlations with peak Vr in systole (Vr-sys, r = 0.5229, p = 0.0001) and in diastole (Vr-dia, r = -0.4870, p = 0.0001). Peak E1 was moderately associated with Vr-sys (r = 0.5087, p = 0.0001) and Vr-dia (r = -0.4771, p = 0.004). Peak twist was moderately correlated with peak VΦ in diastole (VΦ-dia, r = -0.4417, p = 0.0012). See figures 1 and 2.

## Conclusions

DENSE and TPM can provide correlated clinical metrics for quantitatively characterizing regional myocardial motion from different technical aspects.

